# Case of Inflammation in the Mucous Membrane of the Alimentary Canal, and of Malformation of the Heart

**Published:** 1815-08

**Authors:** James Jackson


*For the London Medical and Physical Journal.*

*Case of Inflammation in the Mucous Membrane of the Alimentary Canal, and of Malformation of the Heart;* by JAMES JACKSON, M.D.

THE daughter of R. H. died in Sept. 1813, in her third year. She had been sick five days. Her symptoms were at first vomiting and purging. The vomiting ceased after the use of some remedies, but the purging continued. The stools were exceedingly black and glutinous. They were also very numerous and not inconsiderable in quantity. At the last they became less frequent and changed in colour to green. The child did not suffer much pain. The pulse were greatly accelerated in the beginning and the skin was very hot. She lay quiet through most of the disease, and was even disposed to stupor. Meanwhile the countenance was not very morbid and was of a good colour.

The respiration was most uncommonly accelerated and with peculiar irregularity, as if from being hurried, especially when the pulse were most frequent. The action of the heart was rapid, troubled, and confused. The unusual palpitation of the heart had long and often been noticed by those about the child, as likewise the shortness and rapidity of respiration. It had been particularly observed that this rapidity of respiration was much increased by exercise. The child always manifested an unwillingness to go up stairs, always went up very slowly and often begged to be carried up. She had never been subject to such paroxysms of dyspnœa as seemed to those about her to constitute disease; and it was not remembered that her countenance or extremities had ever been been livid, or even otherwise than fair and florid. Accordingly, medical advice had never been asked in respect to the palpitation and dyspnœa.

The change from life to death was rather sudden.

The cavities of the thorax and abdomen were examined after death, and the following appearances were noticed.

The abdomen was first examined. The viscera did not present any external appearances which are very unusual. The small intestines were, however, more red than usual, but the peritonæum was sound.

The stomach and intestines were laid open. The stomach had its mucous membrane of a bright red colour and swelled in every part. This organ was much contracted and contained a small quantity of thin bloody mucous smeared over its whole surface. The small intestines were inflamed in many places more slightly than in the stomach. The valve of the colon was considerably inflamed, and so was the mucous membrane of the large intestines through their whole extent. This membrane was not only red ; it was also swollen and its surface was rough. It was every where covered with an adhesive mucus. The contents of the intestines were various in colour and considerable in quantity. In one part of the small intestines the colour was yellow, in another part it was green, and in another and this through a considerable portion it was almost precisely that of wet clay. It was however remarkable that these contents were not, in any part, of the dark black colour which had been seen in so many of the stools. This colour had probably been derived from a mixture of the bilious stools with blood poured out from the mucous membrane of the large intestines, and this had ceased before death, so that the last stools were green, as has been mentioned.

The thorax was next examined. The lungs were natural. The heart was uncommonly large. The pericardium was healthy in its appearance, but contained in its cavity more fluid than usual. The auricles were very greatly distended. The great arteries were considerably different from what is common. The pulmonary artery was by much the largest and most prominent. It formed a small arch, which made it appear like the aorta. It divided into two branches immediately as it began to descend after having made its curvature. Of these branches that to the left side was the largest. They went to the lungs as usual ; but from the left branch went off a small vessel to the left subclavian. The aorta rose almost perpendicularly from the heart, passing on the right of the arch of the pulmonary artery ; and after rising rather more than two inches it divided into two branches, which which immediately subdivided into the carotids and subclavians. When the heart was laid open it was found that the *foramen ovale* had not closed, and that there was *an opening between the two ventricles* large enough to allow a finger to pass through it. All the cavities contained coagulated blood, and this was very dark coloured in them all.

Symptoms of dysentery were wanting, although the mucous membrane of the large intestines was in a state of inflammation. But there was probably blood effused; and if the patient had lived longer, the symptoms would have become more truly dysenteric. Death was no doubt accelerated by the malformation of the heart. This had not occasioned any very great difficulty in carrying on the ordinary functions in health, and it is remarkable that it had not; but, when a disease occurred, under which the circulation was quickened, the embarrassment produced in the functions of the heart was such as to occasion great difficulty in all the other functions.

---

